# Deep brain stimulation of the subthalamic nucleus preferentially alters the translational profile of striatopallidal neurons in an animal model of Parkinson's disease

**DOI:** 10.3389/fncel.2015.00221

**Published:** 2015-06-09

**Authors:** Naomi P. Visanji, Iman Kamali Sarvestani, Meaghan C. Creed, Zahra Shams Shoaei, José N. Nobrega, Clement Hamani, Lili-Naz Hazrati

**Affiliations:** ^1^Morton and Gloria Shulman Movement Disorders Centre and the Edmund J. Safra Program in Parkinson's disease, Toronto Western HospitalToronto, ON, Canada; ^2^Faculty of Medicine, Tanz Centre for Research in Neurodegenerative Diseases, University of TorontoToronto, ON, Canada; ^3^Department of Neuroscience, Stockholm Brain Institute, Karolinska InstituteStockholm, Sweden; ^4^Behavioural Neurobiology Laboratory, Campbell Family Mental Health Research Institute, Centre for Addiction and Mental HealthToronto, ON, Canada; ^5^Division of Neurosurgery, Toronto Western Hospital, University of TorontoToronto, ON, Canada

**Keywords:** Parkinson's disease, deep brain stimulation, subthalamic nucleus, striatal medium spiny neurons, translational profile

## Abstract

Deep brain stimulation targeting the subthalamic nucleus (STN-DBS) is an effective surgical treatment for the motor symptoms of Parkinson's disease (PD), the precise neuronal mechanisms of which both at molecular and network levels remain a topic of debate. Here we employ two transgenic mouse lines, combining translating ribosomal affinity purification (TRAP) with bacterial artificial chromosome expression (Bac), to selectively identify changes in translational gene expression in either Drd1a-expressing striatonigral or Drd2-expressing striatopallidal medium spiny neurons (MSNs) of the striatum following STN-DBS. 6-hydroxydopamine lesioned mice received either 5 days stimulation via a DBS electrode implanted in the ipsilateral STN or 5 days sham treatment (no stimulation). Striatal polyribosomal RNA was selectively purified from either Drd2 or Drd1a MSNs using the TRAP method and gene expression profiling performed. We identified eight significantly altered genes in Drd2 MSNs (Vps33b, Ppp1r3c, Mapk4, Sorcs2, Neto1, Abca1, Penk1, and Gapdh) and two overlapping genes in Drd1a MSNs (Penk1 and Ppp1r3c) implicated in the molecular mechanisms of STN-DBS. A detailed functional analysis, using a further 728 probes implicated in STN-DBS, suggested an increased ability to receive excitation (mediated by increased dendritic spines, increased calcium influx and enhanced excitatory post synaptic potentials) accompanied by processes that would hamper the initiation of action potentials, transport of neurotransmitters from soma to axon terminals and vesicular release in Drd2-expressing MSNs. Finally, changes in expression of several genes involved in apoptosis as well as cholesterol and fatty acid metabolism were also identified. This increased understanding of the molecular mechanisms induced by STN-DBS may reveal novel targets for future non-surgical therapies for PD.

## Introduction

Parkinson's disease (PD) is a common neurodegenerative disease characterized by bradykinesia, akinesia, rigidity, and tremor at rest (Lang and Lozano, 1998). It is well established that loss of midbrain dopaminergic neurons in the substantia nigra pars compacta (SNc) is the key pathology underlying the motor deficits of the disease (Forno, 1996). The striatum is the most prominent recipient of these dopaminergic projections and is known to play a key role in mediating the clinical symptoms of PD. The classical model of PD pathophysiology exploits the opposing effects of dopamine on striatopallidal (Drd2) and striatonigral (Drd1a) medium spiny neurons (MSNs) to explain the clinical features of bradykinesia, rigidity, and akinesia (Albin et al., 1989). This model suggests that the loss of striatal dopamine causes an imbalance between the activity of Drd1a and Drd2 populations leading to abnormal activity in recipient neurons in the pallidum and substantia nigra pars reticulata (SNr) leading to abnormal activity in downstream thalamo-cortico-thalamic activity.

Deep brain stimulation targeting the subthalamic nucleus (STN-DBS) is a well-established, increasingly common, surgical treatment to alleviate the motor symptoms of PD. Three of the four cardinal features of PD, rigidity, bradykinesia and rest tremor, are consistently improved by STN-DBS. The magnitude of benefit has been estimated to be ~80% for tremor, and ~40–60% for rigidity, and bradykinesia (Fasano et al., 2012). Furthermore, a ~50% reduction in the dose of dopaminergic medications is feasible post STN-DBS, thereby improving the incidence of dyskinesias (Fasano et al., 2012). The mechanisms through which STN-DBS reduces PD symptoms are a matter of dispute. However, it is clear that STN-DBS results in significant changes in the activity of the entire subthalamo-pallidal loop as well as antidromic effects targeting upstream structures such as cerebral cortex (Li et al., 2007; Hammond et al., 2008). Although a very effective treatment strategy for PD, there are several limitations that drive the search for alternative therapies to STN-DBS. There are obvious risks associated with installing electrodes in the brain. Moreover, levodopa-resistant axial signs, cognitive dysfunction and severe mood or psychiatric disorders can worsen in some individuals following STN-DBS (Follett, 2004; Fasano et al., 2012, 2015). A better understanding of the molecular mechanisms induced by STN-DBS may reveal novel targets for future non-surgical therapies able to selectively reduce the motor symptoms of PD.

Although the striatum is the main input structure of the basal ganglia, receiving substantial input from the cerebral cortex, thalamus and midbrain dopaminergic neurons, and the hallmark symptoms of PD have been explained as an imbalance between Drd2 and Drd1a activity, these striatal neurons are absent from current models explaining the mechanisms of therapeutic effects in STN-DBS (Kopell et al., 2006; Montgomery and Gale, 2008). Here we use microarray technology to describe gene expression changes in both Drd2 and Drd1a MSNs following STN-DBS in a mouse model of PD. Using two BacTRAP transgenic mice expressing GFP under the expressional control of Drd1a and Drd2 dopamine receptor promotors, we are able to detect changes caused by STN-DBS in Drd1a and Drd2 populations separately (Heiman et al., 2008). We demonstrate significant changes in the expression of several genes in Drd2 MSNs with weaker involvement of the Drd1a population following STN-DBS. Our data provide novel insight into the effects of STN-DBS on multiple molecular signaling pathways in striatal MSNs potentially revealing novel targets for development of new therapies targeted at treating the motor symptoms of PD and harnessing the success of STN-DBS by a non-surgical means.

## Materials and methods

### BacTRAP mice

The use of all animals adhered to the humane standards set by the Canadian Council on Animal Care (CCAC). In addition, all experimental procedures have already undergone peer review for scientific merit and a requisite Animal Use Protocol has been approved by the Animal Care Committees at the University of Toronto and Centre for Addiction and Mental Health. The number of animals in each group was restricted to three for humane reasons as the experimental intervention (stereotaxic lesion of the nigrostriatal pathway, followed by stereotaxic implantation of a STN-DBS electrode), was very invasive.

Two Bac transgenic mouse lines were obtained from the Rockefeller Institute. Both lines expressed an EGFP-L10a fusion protein under the control of the dopamine receptor Drd1a (line CP73) or Drd2 (line CP101) promoter. For a full description of the mice please refer to (Doyle et al., 2008; Heiman et al., 2008). Both lines were on a C57BL/6J/Swiss-Webster background and were maintained as transheterozygous.

### 6-OHDA lesion of the median forebrain bundle

Six Drd1a and six Drd2 BacTRAP mice were rendered parkinsonian using the neurotoxin 6-OHDA to create a lesion of the nigrostriatal pathway (Cenci and Lundblad, 2007). All animals were lesioned at 35 days of age. Thirty minute prior to lesioning animals received desipramine (25 mg/kg) and pargyline (5 mg/kg) i.p (Both Sigma Aldrich). Briefly, under general anesthesia, 6-OHDA (3 μg in 0.6 μl) (Sigma Aldrich) was infused unilaterally into the medial forebrain bundle (MFB) at a flow rate of 0.2 μl/min at the following coordinates from Bregma: AP −1.2, ML −1.1, DV −5.0 mm according to the atlas of Paxinos and Franklin (2004). Post-surgery, animals were allowed to recover for a period of 14 days.

### STN DBS electrode implant

Fourteen days post-6-OHDA lesion, all 6-OHDA-lesioned Drd1a and Drd2 BacTRAP mice were unilaterally implanted with STN electrodes in the same hemisphere as the 6-OHDA lesion as described below. Animals were anesthetized with ketamine/xylazine (75/10 mg/kg i.p.) and had their heads fixed to a stereotactic frame. Electrodes with 0.125 μm diameter were connected to a plastic pedestal (Plastics One) and unilaterally implanted in the STN (at the following coordinates from Bregma AP: −1.70 mm, ML: 1.52 mm and from dura DV: −4.5 mm according to the atlas of Paxinos and Franklin (2004) and used as cathodes A screw implanted over the somatosensory cortex was used as the anode. Two additional screws were attached to the skull for better securing the cap in place. Electrodes and screws were fixed to the skull with dental acrylic cement. Supplemental Figure 1 illustrates the location of the electrode tip within the STN.

### Deep brain stimulation protocol

Stimulation was conducted with a handheld stimulator (ANS model 3510) at 100 μA, 130 Hz, and 90 μs for 4 h/day for 5 consecutive days. This current was chosen as, based on the configuration of our electrodes, it generates a charge density comparable to that used in patients with DBS electrodes and should therefore be in a safe range not to create a lesion (Hamani et al., 2010; Hamani and Nobrega, 2012). The selected pulse width and frequency are similar to those used in clinical practice. Sham treated parkinsonian animals had electrodes implanted in the STN but did not receive stimulation.

### Post-mortem verification of lesion efficiency

Post mortem, lesion efficiency was assessed by calculating nigral tyrosine hydroxylase (TH) immunoreactive cell loss. Animals were euthanized immediately the stimulator was turned off following 5 consecutive days of stimulation. The brain was removed and dissected. The midbrain, including SNc was immersion fixed in 4% paraformaldehyde. Following fixation in 4% paraformaldehyde, blocks encompassing the entire midbrain were paraffin embedded and 5 μ-thick serial sections taken from −3.08 and −3.28 mm relative to Bregma according to the atlas of Franklin and Paxinos (2007). Briefly, endogenous peroxidase was blocked with 3% hydrogen peroxide, antigen retrieval was with 10 mM citrate buffer at pH 6.0 and sections were stained overnight with rabbit polyclonal antibody to TH (Novus Biologicals) at 1/1500 dilution. Staining was finished with Vector's Peroxidase ImmPRESS detection system, color developed by DAB and sections were counterstained with Mayer's hematoxylin prior to coverslipping. TH positive cells were defined as those with a brown cell membrane with distinct lighter rounded cell body. All animals included in the study had a >90% loss of TH immunoreactive cells in the lesioned hemisphere as compared to the intact hemisphere (data not shown).

### Translating ribosomal affinity purification (TRAP)

The rostral portion of the brain, including striatum, was dissected and translational polyribosomal RNA was isolated from the lesioned hemisphere via the TRAP technique using the polysome purification protocol provided by BacTRAP.org (http://www.bactrap.org/downloads/Polysome_IP_Protocol.pdf). All steps were carried out under RNAse free conditions. Dissected striata were individually homogenized, using a motor driven teflon-glass homogenizer, in ice cold lysis buffer (20 mM HEPES KOH, 5 mM MgCl2, 150 mM KCL, 0.5 mM DTT, 100 μg cyclohexaminde, 40 U/ml Rnasin and protease inhibitor cocktail, pH 7.4). The homogenates were then centrifuged at 2000× g for 10 min at 4°C. Homogenates were then mixed in NP-40, final concentration of 1%, and 1,2-diheptanoyl-sn-glycero-3-phosphocholine (DHPD, Avanti Polar Lipids, Alabaster, AL), final concentration of 30 mM for 5 min before centrifugation at 20,000 × g for 10 min at 4°C. Homogenates were then immunoprecipitated for 30 min at 4°C with end-over-end mixing with 200 μl freshly-prepared antibody-bound beads [50 μg each of anti-GFP antibodies (clone 19C8 and 19F7, Memorial Sloan-Kettering Monoclonal Antibody Facility) were bound to Dynal (protein G magnetic beads, Invitrogen, Carlsbad, CA) by incubation with slow end-over-end mixing for 1 h at room temperature]. Following incubation, beads were collected using a magnetic rack then washed four times in ice cold wash buffer 20 mM HEPES-KOH, 5 mM MgCl_2_, 350 mM KCL, 0.5 mM DTT, 1% NP-40, 100 μg cyclohexaminde, pH 7.4. After the last wash, RNA was eluted from the beads according the manufacturer's instructions using an Absolutely RNA nanoprep kit (Stratagene, La Jolla, CA) with in column DNA digestion. RNA quantity was determined using a Nanodrop 1000 spectrophotometer (Wilmington, DE).

### Microarray

RNA quality and integrity were assessed using a bioanalyzer 2100 (Agilent) prior to microarray. For microarray 1 ng of each sample was amplified (Nugen) and labeled using Illumina totalprep 96-rna amplification kit (Ambion). 1.5 μg of cRNA of each sample generated from the amplification kit was hybridized into one mouse wg-6 v2.0 beadchip (Illumina). The beadchip was incubated at 58°C rotating for 18 h for hybridization. The beadchip was then washed and stained as per Illumina protocol and scanned on the iscan (Illumina). Data files were quality control tested and quantified in genomestudio version 2010.1 (Illumina). Data was analyzed using Genespring and r/Bioconductor software.

### Data analysis

**Figure 1:**
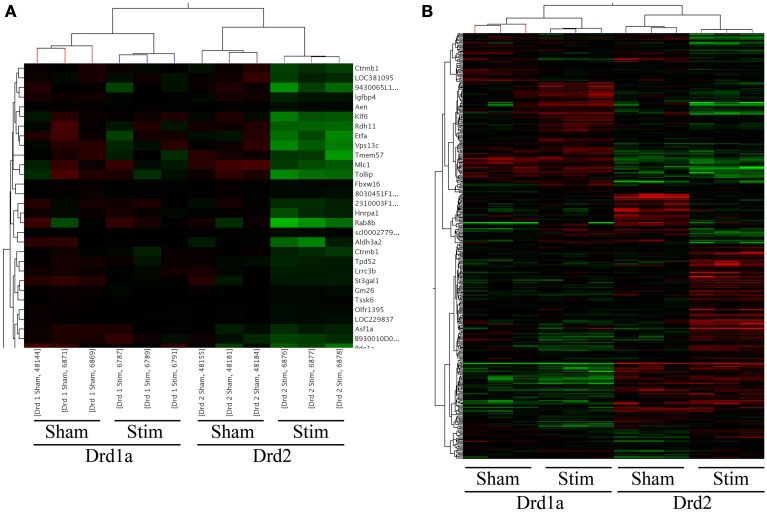
**Heat-maps of supervised clusters of gene expression changes in Drd1a and Drd2 expressing MSNs following STN-DBS**. Probes consistently altered in correlation are clustered together. **(A)** A magnification of the clustering results showing altered genes (rows) by sample (columns). The dendogram on top illustrates the similarity of samples within each group, whereas the dendogram to the left shows the hierarchical clustering based on similarity between the expression of genes. **(B)** Heat map illustrating array-wide gene expression between the four experimental groups. A clear separation in the gene expression between Drd1a and Drd2 expressing MSNs is apparent as well as changes in expression of several genes following STN-DBS. Changes in gene expression following STN-DBS are best observed in Drd2 MSNs. Except for genes on the top of the figure, expression of most genes remain almost unchanged in Drd1a MSNs.

Data was imported in GeneSpring v12.1 for analysis. During import, the data was normalized using a standard (for Illumina arrays) quantile normalization followed by a “per probe” median centered normalization. All data analysis and visualization were performed on log2 transformed data. This study comprised 4 experimental groups (Drd1a sham, Drd1a STN-DBS, Drd2 sham, and Drd2 STN-DBS). A total of 45281 probes are represented on the Mouse WG-6 V2 BeadChip. Data was first filtered to remove the confounding effect probes that show no signal may have on subsequent analysis. Only probes that were above the twentieth percentile of the distribution of intensities in 100% of any of the one of four above groups were allowed to pass through this filtering. The final set contained 37,650 probes. An unsupervised clustering using a Pearson centered correlation as a distance metric with average linkage rules in the tree building algorithm of this set of probes demonstrated a reasonable, but not perfect, separation between the samples into the experimental groupings (Figure 1).

### Quantitative real time polymerase chain reaction

Microarray data were validated using real time quantitative real time polymerase chain reaction (qRT-PCR) of selected transcripts showing a differential expression in DBS and Sham animals. Primer pairs were designed using Primer3 software (http://bioinfo.ut.ee/primer3/). 2μg of total RNA sample were reverse transcribed using Superscript III reverse transcriptase (Invitrogen) and the diluted cDNA (equivalent to 100 ng of total RNA) underwent qRT-PCR in triplicate using SYBR Premix Ex Taq (Perfect Real Time) (TaKaRa) and 7500 Realtime PCR System (Applied Biosystems) according to the manufacturers' instructions. In all cases β-Actin (Actb) served as an internal control.

## Results

This pilot study included animals in four experimental groups. All animals had a 6-OHDA lesion of the median forebrain bundle. The sample groups comprised of Drd1a mice receiving STN-DBS, Drd1a mice implanted without stimulation (sham), Drd2 mice receiving STN-DBS and Drd2 sham mice (see Materials and Methods). We performed two One-Way ANOVA tests with Benjamini-Hochberg False Discovery Rates of *p* < 0.1 (corrected) and *p* < 0.01 (uncorrected) between all sample groups. These tests yielded 16 and 728 significantly varying probes listed in Supplementary Tables 1 and 2, respectively. The uncorrected probes were clustered to reveal genes altered similarly (Figure 1). The two right columns of Figure 1B show that expressions of many Drd2 MSN genes have changed due to STN-DBS. In comparison, expressions of much fewer Drd1a genes change following DBS (the two left columns in Figure 1B).

The 728 genes from the uncorrected One-Way ANOVA were next filtered to remove genes with a <1.5 fold change. Out of 728 uncorrected genes, expression of only 291 genes altered greater than 1.5 folds in at least one of the four comparisons. Interestingly, expression of 102 genes changed specifically in Drd1a MSNs while expression of only 56 genes altered in Drd2 MSNs following STN-DBS. Expression of 14 genes shared between Drd1a and Drd2 genes changed following the stimulation.

We are specifically interested in two major pairwise comparisons: genes altered in Drd1a (Drd1a sham vs. Drd1a stim.) and Drd2 (Drd2 sham vs. Drd2 stim.) MSNs following STN-DBS. Two minor comparisons i.e., genes differentially expressed between Drd1a and Drd2 MSNs before (Drd1a sham vs. Drd2 sham) and after (Drd1a stim. vs. Drd2 stim.) stimulation are also reported to add to the prior knowledge about genetic differences between Drd1a and Drd2 MSNs in health and disease (Heiman et al., 2008, 2014; Visanji et al., 2012; Visanji et al., submitted).

To perform specific pairwise comparisons of interest, we applied a *post-hoc* Tukey's honest significant difference test (THSD) to the 291 uncorrected filtered genes. Finally a Two-Way ANOVA (uncorrected) was also performed with MSN type (Drd1a or Drd2) and treatment (STN-DBS or sham) as the factors (Supplementary Tables 3, 4). Supplementary Tables 5, 6 list genes involved in the two major pairwise comparisons while Supplementary Tables 7, 8 list those of the two minor comparisons. Each of Supplementary Tables 5–8 contains four sublists of genes, each representing the results of corresponding statistical tests performed in order of stringency: One-Way ANOVA (corrected), One-Way ANOVA (uncorrected passing *post-hoc* THSD), Two-Way ANOVA (uncorrected) and uncorrected results failing THSD (listed in black, blue, red, and green, respectively).

**Figure 2:**
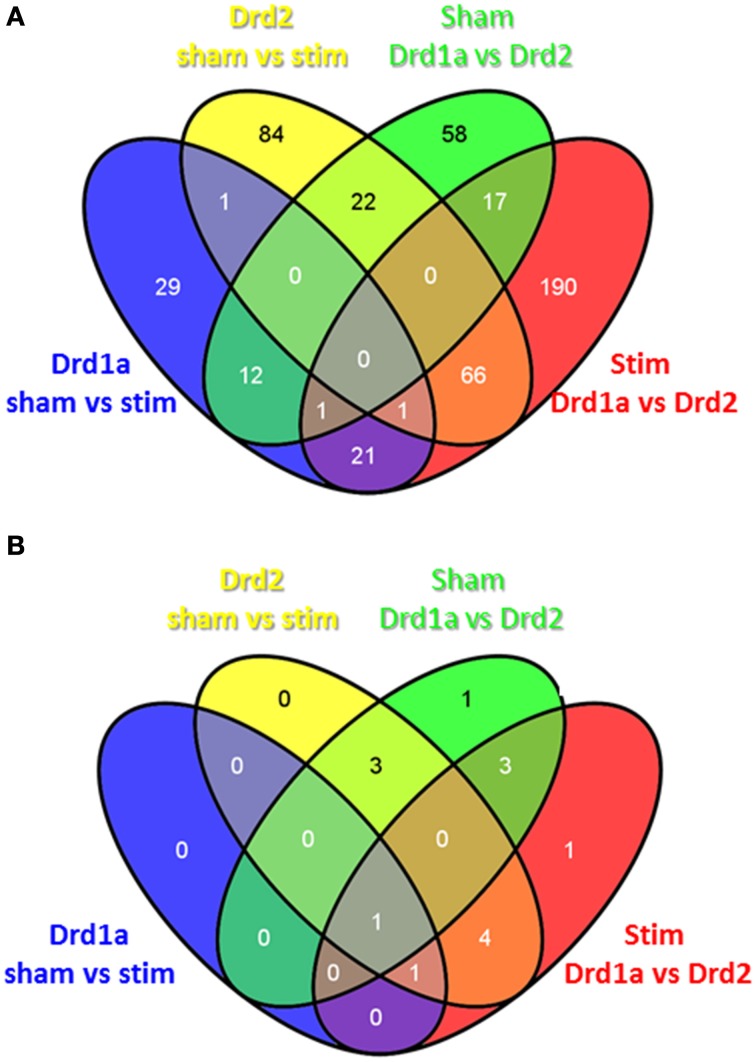
**Overlap of differentially expressed genes in the direct and indirect pathways following STN-DBS in a mouse model of PD. (A)** Uncorrected **(B)** Corrected. All genes underwent a filtering process to remove those exhibiting <1.5 fold change. Drd1a Sham vs. stim: Genes altered in Drd1a MSNs before and after STN-DBS. Drd2 sham vs. stim: Genes altered in Drd2 MSNs before and after STN-DBS. Sham Drd1a vs. Drd2: Genes differentially expressed between Drd1a and Drd2 MSN before STN-DBS. Stim Drd1a vs. Drd2: Genes differentially expressed between Drd1a and Drd2 MSN after STN-DBS.

Venn diagrams were used to demonstrate which gene expression changes were common to and exclusive to each of the different experimental conditions. Figure 2A demonstrates that out of 285 and 197 genes altered after STN-DBS in Drd2 and Drd1 MSNs respectively, 102 genes are shared. 183 genes exclusively alter in Drd2 MSNs, and 95 genes alter exclusively in Drd1a MSNs. This observation suggests that the major influence of STN-DBS on striatal MSNs is exerted on Drd2 MSNs with only minor involvement of the Drd1a population. This pattern is also evident when considering the corrected genes alone (Figure 2B), thus following STN-DBS there are eight significantly altered corrected genes in Drd2 MSNs with two of these genes also significantly altered in Drd1a MSNs.

### Functional analysis

The eight corrected genes exhibiting a >1.5 fold change in Drd2 MSNs after STN-DBS (Vps33b, Ppp1r3c, Mapk4, Sorcs2, Neto1, Abca1, Penk1, and Gapdh) and two overlapping candidate genes in Drd1a MSNs (Penk1 and Ppp1r3c) were used as backbones for functional analysis to create a framework to exploit uncorrected results of changes in Drd1a and Drd2 MSNs following STN-DBS (Table 1). Thus, eight functional clusters were generated by assigning each uncorrected gene to one of the functions defined by corrected genes. The first functional cluster, built upon a significant change (corrected) in Ppp1r3, Neto1, and Sorcs2 contains 22 genes and describes genes related to dendritic excitability (Supplementary Table 9 sheet 1). Since Ppp1r3 also affects somatic excitability, a second cluster with 29 members was formed around Ppp1r3c (Supplementary Table 9 sheet 2). A third functional cluster, built upon a significant change (corrected) in MapK4 describes 53 genes related to synaptogenesis (Supplementary Table 9 sheet 3). The fourth cluster describes genes related to the fusion of vesicles and is built upon a significant change (corrected) in Vps33B and Ppp1r3c and contains 24 genes (Supplementary Table 9 sheet 4). In line with their role in adjusting dendritic excitability, both Ppp1r3 and Neto1 are involved in calcium influx to the MSNs. Therefore, a separate cluster describing genes related to Calcium metabolism which contains 25 genes was formed (Supplementary Table 9 sheet 5). The sixth cluster containing 11 genes built upon a significant change (corrected) in Abca1 describes genes related to cholesterol and very long chain fatty acid metabolism (Supplementary Table 9 sheet 6). The seventh cluster is based on significant (corrected) change in Penk1 following STN-DBS in both Drd1a and Drd2 expressing MSNs (Supplementary Table 9 sheet 7). This cluster is related to the changes in neuropeptides and monoamines which serve a very broad range of functions. The final and largest cluster containing 58 genes built upon a significant change (corrected) in three genes (Ppp1r3c, Sorcs2, and Gapdh) describes genes related to apoptosis (Supplementary Table 9 sheet 8).

**Table 1:** **Fold changes of significantly altered genes (corrected) in either Drd2 or Drd1a expressing MSNs following STN-DBS and associated functional clusters for functional analysis**.

**Functional cluster**	**Significant gene**	***P* value**	**Drd2 MSNs**	**Drd1a MSNs**
			**Fold change (STN-DBS vs. sham)**	**Fold change (STN-DBS vs. sham)**
Dendritic excitability	Ppp1r3c	2.17E-05	3.966	−2.008
	Neto1	2.97E-05	−2.364	−1.049
	Sorcs2	2.53E-07	−2.415	−1.038
Somatic excitability	Ppp1r3c	2.17E-05	3.966	−2.008
Synaptogenesis	MapK4	3.75E-05	−3.061	1
Fusion of vesicles	Vps33B	4.84E-07	4.441	1.378
	Ppp1r3c	2.17E-05	3.966	−2.008
Calcium metabolism	Ppp1r3c	2.17E-05	3.966	−2.008
	Neto1	2.97E-05	−2.364	−1.049
Cholesterol and VLCFA metabolism	Abca1	9.15E-06	1.724	−1.067
Neuropeptides and monoamines	Penk1	3.37E-05	−1.59	1.523
Apoptosis	Gapdh	2.76E-05	−1.537	1.1273
	Sorcs2	2.53E-07	−2.415	−1.038

Further clusters were also generated by grouping functionally related uncorrected genes only. Comprehensive lists of genes involved in each major uncorrected function/cluster are given in Supplementary Table 9 (sheets 9–20).

### Validation of findings

qRT-PCR was performed on five selected genes to verify the microarray results. We chose four marker genes Drd1a, Drd2, Pdyn, and Penk1 as well as Neto1 as a significantly altered gene after STN-DBS. Results of the qRT-PCR are in accordance with the microarray results. Thus, expression of Pdyn decreases in Drd1a MSNs and expression of Penk1 increases in Drd2 MSNs while expression of Drd1a and Drd2 remain constant following DBS. Moreover, STN-DBS leads to an increase in level of Neto1 gene in Drd2MSNs but no change in Drd1a MSNs.

## Discussion

**Figure 3:**
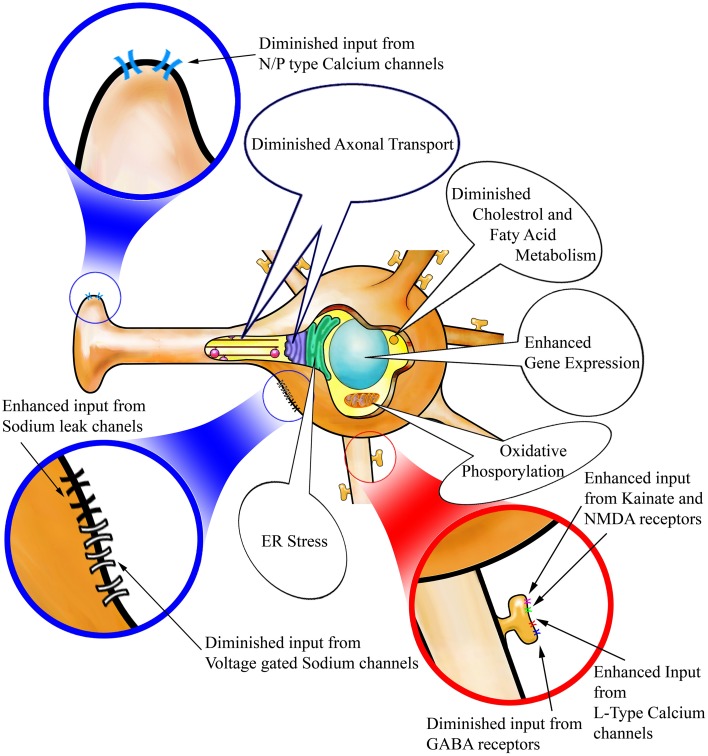
**Alterations in cellular mechanisms in Drd2-expressing striatopallidal MSNs following STN-DBS**. STN-DBS changes the expression of genes involved in spine formation and synaptogenesis. These changes suggest that more spines and synapses may be formed on Drd2 MSNs after stimulation. In addition, **(A)** Excitatory input to the spines through kainate and NMDA receptors as well as L-Type Calcium channels is enhanced and inhibitory input via GABA receptors is suppressed so that the neuron may become more excitable on it dendritic input. **(B)** Voltage gated Sodium channels and Sodium leak channels are suppressed thus potentially rendering the neurons less prone to action potential generation. **(C)** N/P type calcium channels are suppressed on the axon terminals potentially hindering vesicular release. Axonal transport machinery is suggested to be diminished in Drd2 MSNs accordingly. Other changes observed in Drd2 MSNs suggest high stress induced in these neurons due to STN-DBS. Such changes are related to Oxidative and ER stress and abnormal lipid and cholesterol metabolism and apoptosis. Such changes may be results of enhanced input and diminished output to these neurons.

Our findings demonstrate that the most striking effects of STN-DBS on striatal MSNs are exerted on the Drd2 expressing population of the indirect pathway with only minor changes apparent in Drd1a expressing MSNs of the direct pathway. Thus, within the indirect pathway there are eight significantly altered genes following STN-DBS. Of these eight genes, two were also significantly altered in the direct pathway, but no genes were significantly altered in exclusively in the direct pathway. A complementary study may also investigate the temporal changes in gene expression during the 14 days of nigro striatal degeneration. The observed dominant involvement of the indirect pathway is unsurprising as both the striatofugal and subthalamofugal projections are highly collateralized systems. A predominant involvement of the indirect vs. the direct pathway may be explained by these collateralized systems. However, it is established that corticosubthalamic axons are collaterals of corticospinal axons which also send collaterals to MSNs of the indirect pathway with only minor innervation of the MSNs of the direct pathway (Reiner et al., 2010; Kita and Kita, 2012). Furthermore, this finding supports the suggestion that the antidromic activation of corticosubthalamic neurons is involved in the therapeutic effect of STN-DBS (Li et al., 2007; Hammond et al., 2008). It has been largely debated if STN-DBS causes excitation or inhibition in STN and its downstream targets in pallidum (Kopell et al., 2006; Montgomery and Gale, 2008). The major genetic changes we observe in the striatum are not likely to be a result of long polysynaptic loops linking the subthalamic nucleus to the striatum such as STN-GPi-Thalamo-Cortico-Striatal circuit because each synapse is facilitated with several mechanisms to dampen hyper- and hypo-excitation. Our results however, show a pronounced over activity in striatum which may be best described in the light of antidromic activity of motor cortical neurons following STN-DBS. In concert, our observations suggest that, by various mechanisms discussed in details below, following STN-DBS the indirect pathway is capable of receiving an enhanced input; however concurrent alterations are also apparent that would dampen the output of these Drd2 MSNs. The combined effects of STN-DBS on MSNs of the indirect pathway are summarized in Figure 3. Our discussion will focus on each of the cellular mechanisms implicated in this imbalance between the input and output of striatal MSNs.

## Mechanisms of enhanced input

### Enhanced dendritic excitability

Expression of Ppp1r3c is decreased 3.97 fold in Drd2 MSNs post STN-DBS. Ppp1r3c is a regulatory subunit of protein phosphatase-1 (PP1) which plays a key role in inhibition of protein kinase A (PKA). PKA and PP1 compete to modulate ion channels in opposing directions. Thus, while most cation channels on MSN membranes (AMPA, NMDA, L-type Ca) are upregulated by PKA and downregulated by PP1, most anion channels (GABA) are downregulated by PKA and upregulated by PP1. Two major exceptions are N/P-type calcium channels, involved in release of vesicles from terminal boutons, and fast voltage gated Na channels, involved in generation of action potentials, both which are downregulated by PKA and upregulated by PP1. Decreased expression of Ppp1r3c in the indirect pathway following STN-DBS may therefore result in an increased excitatory inflow to Drd2 MSNs via increased cationic vs. anionic influx.

Neto1 is an auxiliary subunit of kainaite receptors (KARs) purportedly responsible for the slow kinetics and high affinity of GluK2 subunit of these receptors (Molnar, 2013) which is the dominant subunit in the striatum. In addition to KARs, Neto1 is also associated with NMDARs via an intracellular domain interaction with GluN2A and GluN2B subunits (Cousins et al., 2013). Neto1 is reported to be required for proper delivery and stability of NMDARs containing GluN2A subunit to the postsynaptic density and knockout mice lacking this gene show impaired spatial learning (Ng et al., 2009). Via its interaction with KARs and NMDARs, Neto1 is located in an ideal location to control the excitatory input to the MSN. Expression of this gene increases in Drd2 MSNs 2.36 fold but remains stable in Drd1a MSNs following STN-DBS. Thus, STN-DBS may enhance the activity of KARs and NMDARs on Drd2 expressing MSNs, a result that is in accordance with our suggestion that STN-DBS increases dendritic excitation in the indirect pathway.

The hypothesis that STN-DBS modifies dendritic excitability is further supported by uncorrected changes in 28 genes in Drd2 expressing MSNs and 13 Genes in Drd1a MSNs (Supplementary Table 9 Sheet 1). Some of the altered genes directly code for ion channels or their accessories, while others are modulators of ion channels. A variety of protein kinases and phosphatases which are known to modulate activity of voltage and ligand gated ions channels on MSNs are also among the significantly altered genes.

### Dendritic spine formation

Expression of Mapk4 (Erk4) increases 3.06 fold in Drd2 MSNs but remains stable in Drd1a MSNs following STN-DBS. Erk4 is preferentially expressed in the prefrontal cortex, olfactory bulb and the striatum (Rousseau et al., 2010). Erk4, and paralogous Erk3 (Mapk6), use MK5 as a substrate to affect gene expression in cells and the Erk3-MK5 signaling complex has been shown to induce spine formation on hippocampal neurons (Brand et al., 2012). Thus, Mapk4 may also be involved in the process of dendritic spine formation in striatal MSNs. The increased expression of Erk4 three in Drd2 MSNs following STN-DBS may preferentially induce the formation of dendritic spines in Drd2 expressing MSNs leading to an enhanced ability to receive input in these neurons.

In total, 40 genes involved in neurite outgrowth are altered in Drd2 MSNs and 31 in Drd1a MSNs after STN-DBS suggesting that dendritic morphology may be significantly altered after STN-DBS. The neuron navigator gene Nav1 increases 2.58 fold selectively in Drd1a MSNs after stimulation. Similarly, expression levels of Rarb, encoding retinoic acid receptor beta, which has been shown to induce spine formation (Chen and Napoli, 2008), increases 3.24 fold selectively in Drd2 MSNs. Gene expression of Cadherin 4, a calcium dependent regulator of neurite outgrowth and synaptogenesis, increases 2.4 fold and 1.65 in Drd1a and Drd2 MSNs respectively. Furthermore, there is a 2.99 fold decrease in expression of Cspg5, a negative signal for neurite growth, in Drd1a neurons after DBS with negligible (~9%) change in Drd2 MSNs. Expression of Baiap2, an insulin receptor tyrosine kinase substrate known to regulate spine formation on dendritic branches (Choi et al., 2005) and rac-mediated actin cytoskeleton regulation (Connolly et al., 2005), decreases 2.09 fold in Drd1a MSNs and increases 1.79 fold in Drd2 MSNs. This would have widespread implications for MSN function and certainly warrants further study (Supplementary Table 9 Sheet 3).

### Calcium influx

Both Ppp1r3c and Neto1 are also implicated in calcium influx. As stated above, PP1 inhibits the activity of L-Type calcium channels while the involvement of Neto1 in NMDA receptor activity modulates calcium influx. Furthermore, Neuronatin (Nnat), a dendritically translated gene whose over expression releases calcium from endoplasmic reticulum (Oyang et al., 2011) increases 2.67 fold in Drd1a expressing MSNs after DBS. In total, 14 genes in Drd2 and six genes in Drd1a MSNs all involved in regulation of calcium level in the MSNs are altered significantly after STN-DBS. Altering the neuronal calcium concentration can cause a myriad of effects, including influencing excitability, vesicular release and, in excess, neurotoxicity. Indeed, several of these genes implicated in calcium influx are also implicated in apoptosis, as discussed below.

## Mechanisms of diminished output

### Reduced somatic excitability

Scn2a1, which encodes the alpha subunit of fast sodium channels, is of particular interest in the present study as its pattern of expression shifts from being dominant in the indirect pathway in sham stimulated animals to being dominant in the direct pathway following STN-DBS. Thus, following STN-DBS Scn2a1 increases 2.87 fold in Drd1a MSNs and decreases 2.21 fold in Drd2 MSNs. Similarly, Nalcn which encodes sodium leak channels, decreases 2.46 fold in Drd2 MSNs and 3.35 fold in Drd1a MSNs following STN-DBS. A reduction in sodium leak channels may lead to a hyperpolarized resting potential such that excitatory postsynaptic potentials (EPSPs) are less likely to generate action potentials.

Our results also show mixed patterns regarding expression of voltage gated potassium channels following STN-DBS. While expression of Kcnab1 increases 2.25 fold in Drd2 expressing MSNs, expression of Kcna1 deceases 1.52 fold and increases 1.96 fold in Drd1a MSNs. These results indicate that the shape and the refractory period of the action potential may be altered in Drd1a and Drd2 MSNs after STN-DBS. Future studies employing electrophysiology would be required to elucidate potential changes in action potential characteristics following STN-DBS.

### Reduced transport and fusion of vesicles

Vps33b is a Sec1/Munc18 like gene involved in trafficking of vesicles and their engagement with the SNARE complex and fusion (Gissen et al., 2005; Baker et al., 2013). Vps33b expression decreases 4.44 fold in Drd2 MSNs and only 1.38 fold in Drd1a MSNs post STN-DBS, suggesting that vesiclular trafficking and release may be preferentially diminished in the indirect pathway. Sixteen additional genes are also implicated in transport and fusion of vesicles (Supplementary Table 9 sheet 4) and a further 17 genes involved in axonal cargo transport undergo significant changes after STN-DBS (Supplementary Table 9 sheet 11). For example expression of the kinesin superfamily member, Kif1b is decreased 8.01 fold in Drd2 MSNs and increased 1.58 fold in Drd1a MSNs after STN-DBS, suggesting a hindrance of axonal transport in Drd2 MSNs.

## Other effects of STN-DBS

### Long term plasticity

Several genes associated with long term plasticity are significantly altered following STN-DBS. Sorcs2 is preferentially expressed in the striatum and olfactory bulb and is believed to mediate long term depression (LTD) by acting as a co-receptor for proBDNF. BDNF promotes long term potentiation (LTP) via its interaction with TrkB receptors while proBDNF promotes LTD via interaction with a p75-Sorcs2 complex. Thus, the pro and the mature forms of BDNF are suggested to compete with each other providing means of bidirectional plasticity (Je et al., 2012). Our results indicate that in Drd2 MSNs, Sorcs2 expression is 2.42 fold higher after STN-DBS. Thus, in Drd2 MSNs the competition between LTP and LTD may tip toward LTD following STN-DBS. Expression of Sorcs2 was unchanged in Drd1a MSNs following STN-DBS. Interestingly it has been shown by others that 6-OHDA lesioning of the nigrostriatal pathway leads to a selective loss of dendritic spines in Drd2 expressing MSNs, yet remaining Drd2 spines undergo LTP (Day et al., 2006). Our results suggest that Drd2 but not Drd1a MSNs may tend to recover lost spines but in turn Drd2 MSN spines undergo LTD. Thus, STN-DBS may have the reverse effect of 6-OHDA lesioning reported by Day and colleagues.

### Cholesterol and fatty acid metabolism

In total, eight Drd2 and five Drd1a MSN genes involved in metabolism of cholesterol and fatty acids are significantly altered after STN-DBS. Abca1 mediates the efflux of cholesterol and phospholipids to lipid-poor apolipoproteins (apo-A1 and apoE). It has been shown that the expression of Abca1 is correlated with intensity of dementia in Alzheimer's patients (Akram et al., 2010). Expression of the Abca1 gene decreases 1.72 fold in Drd2 expressing MSNs but remains unchanged in Drd1a MSNs. This suggests that cholesterol may not be efficiently removed from MSNs of the indirect pathway after STN-DBS. Intracellular cholesterol is known to promote synaptogenesis and axonal and dendritic growth (Karasinska and Hayden, 2011). Thus, elevated cholesterol levels in Drd2 expressing MSNs would facilitate the formation of new spines, a process already highlighted by our observed effects of STN-DBS on Mapk4 in Drd2 expressing MSNs. It is important to note that Hdlbp, that encodes high density lipoprotein binding protein and likely functions in the removal of excess cellular cholesterol, decreases 3.12 fold in Drd1a MSNs and increases 2.82 fold in Drd2 MSNs. This observation may point to a compensatory mechanism within Drd2 MSNs in reaction to elevated cholesterol. Further research should elucidate the influence of STN-DBS on striatal cholesterol metabolism.

There was a 5.42 fold decrease in Abcd2 in Drd1a MSNs following STN-DBS. The peroxisomal ATP-binding cassette transporter encoded by this gene is implicated in transport of very long chain fatty acids into peroxisomes. Thus, reduced expression of Abcd2 may suggest diminished fatty acid break down in Drd1a MSNs. Other peroxisome related genes, Pex13 and Pex2 are significantly altered following STN-DBS. Pex2 is decreased 2.44 fold in Drd1a MSNs and increased 1.83 fold in Drd2 MSNs and Pex13 is increased 1.58 fold in Drd2 MSNs after STN-DBS. As opposed to Abcd2, these two genes are not transporters of very long chain fatty acids, instead they are needed for biogenesis of proxisomes. Our data showing that both synthesis of peroxisomes and transport of fatty acids into them is diminished in Drd1a MSNs, suggest that fatty acid accumulation may occur in Drd1a MSNs after STN-DBS. In Drd2 MSNs our findings suggest an increased synthesis of peroxisomal subunits which may be an effort to counteract the observed diminished transport of very long chain fatty acids in Drd2 MSNs. Additionally, Pex genes have been reported to interfere with alpha-synuclein aggregation so it remains possible that DBS may alter further aggregation of alpha-synuclein in PD.

Build-up of very long chain fatty acids can interfere with a variety of striatal signaling mechanisms including endocannabinoid signaling (Lovinger and Mathur, 2012) and diacylglycerol metabolism. The hypothesis that STN-DBS my enhance diacylglycerol in striatal MSNs is further supported by the observation that expression of Dgkq, a gene which encodes a strong inhibitor of diacylglycerol (diacylglycerol kinase), is altered following STN-DBS. Thus, this gene is expressed slightly higher (27%) in Drd1a MSNs compared to Drd2 MSNs prior to stimulation, but following STN-DBS this pattern is reversed such that DGKQ is 2.7 fold higher in Drd2 MSNs than Drd1a MSNs.

### Enkephalin and immediate early genes

It has long been established that Enkephalin is co-expressed with D2 receptors in Drd2 MSNs where it has been suggested to play a regulatory role in expression of immediate early genes (IEGs). Thus, enkephalin inhibits IEGs after they are induced following D2 receptor blockade (Steiner and Gerfen, 1998). Furthermore, expression of the proenkephalin gene, Penk1 increases following 6-OHDA lesions of the nigrostriatal pathway. Our results show that expression of Penk1 also increases a modest 1.59 fold in Drd2 expressing MSNs after STN-DBS, whereas enkephalin convertase, Cpe, decreases 1.8 fold, which would be expected to result in the inhibition of IEGs. The timing of extraction of RNA in the present study precludes the analysis of IEGs, however changes in IEGs would be unlikely to be implicated in the long-term effects of STN-DBS.

### Apoptosis

The largest cluster generated by our functional analysis implies that apoptosis may be induced in Drd2 expressing MSNs post STN-DBS. In total 43 uncorrected genes involved in apoptosis were mapped onto the backbone of a significant increase in Gapdh of 1.54 fold in Drd2 expressing MSNs (Supplementary Table 9 Sheet 8). It has been suggested that Gapdh is involved in apoptotic cascades in neurodegenerative diseases (Chuang et al., 2005). Furthermore, Gapdh is implicated in the cytotoxicity of both mutant Huntingtin (Bae et al., 2006) and Htt (Kaltenbach et al., 2007). Moreover, two other corrected genes Ppp1r3c and Sorcs2 are also implicated in apoptosis and a fourth corrected gene, Nbn, implicated in apoptosis was shown to undergo a subthreshold 1.48 fold change in Drd2 MSNs. A large increase in the expression of Zeb2 in Drd1a MSNs (4.68 fold) and marked decrease in Drd2 MSNs (3.43 fold) is also observed. This gene is a repressor of the TGF beta pathway (Postigo, 2003) and is suggested to inhibit apoptosis in neurons. Collectively these data suggest that STN-DBS may selectively promote apoptosis in Drd2 expressing MSNs. Moreover, Sepp1, which encodes a selenium transport protein and considered as a primary line of defense against oxidative stress, is decreased 4.34 fold in Drd2 MSNs and increased 2.45 fold in Drd1a MSNs following STN-DBS. This would render Drd2 expressing MSNs more susceptible to oxidative stress following STN-DBS. Although human studies have shown profound changes in cell death pathways and in peripheral blood in PD in human and mouse (Macchi et al., 2015), it is not straight forward to expand that pathologic effect to current therapeutic effect. Future studies looking at the number of viable striatal Drd2 expressing MSNs should reveal the extent of this potentially damaging effect of stimulation.

## Conclusions

In conclusion, our data suggest that at the level of the striatum, the influence of STN-DBS is predominantly exerted on the projections neurons of the indirect pathway with only minimal effect on the direct pathway. This is in contrast to changes induced in striatal projection neurons following administration of L-DOPA. Recent studies (Visanji et al., 2012; Heiman et al., 2014) have shown that following L-DOPA administration expression of many more genes change in Drd1a compared to Drd2 MSNs. Interestingly, striatal genes whose expression changes following L-DOPA administration (Visanji et al., 2012; Heiman et al., 2014) have a very slim overlap with the striatal genes whose expression changes following STN-DBS (only three Drd1a genes and three Drd2 genes). This may suggest that mechanisms controlling therapeutic effect of STN-DBS may be very different from those causing the pathology of PD. The combined alterations in gene expression in the indirect pathway on the one hand would lead to generation of larger EPSPs, smaller IPSPs, increased dendritic spines and higher calcium influx into the dendritic tree of these neurons but at the same time processes are implicated that would hamper the process of initiation of action potentials, transport of neurotransmitters from soma to axon terminals and vesicular release. Finally, changes in expression of several genes suggest that apoptosis is promoted in Drd2 expressing MSNs following DBS and that cholesterol and fatty acid metabolism may also be affected by STN-DBS. This increased understanding of the molecular mechanisms induced by STN-DBS will hopefully initiate further investigation of novel targets for future non-surgical therapies to reduce the motor symptoms of PD.

### Conflict of interest statement

The authors declare that the research was conducted in the absence of any commercial or financial relationships that could be construed as a potential conflict of interest.
