# Supplementary material for: Deep brain stimulation of the subthalamic nucleus preferentially alters the translational profile of striatopallidal neurons in an animal model of Parkinson's disease
Source: Front Cell Neurosci. 2015 Jun 9;9:221. doi: 10.3389/fncel.2015.00221 (PMC4460554; doi:10.3389/fncel.2015.00221)
Supplement: Supplementary file 1 [file Image1.PDF]

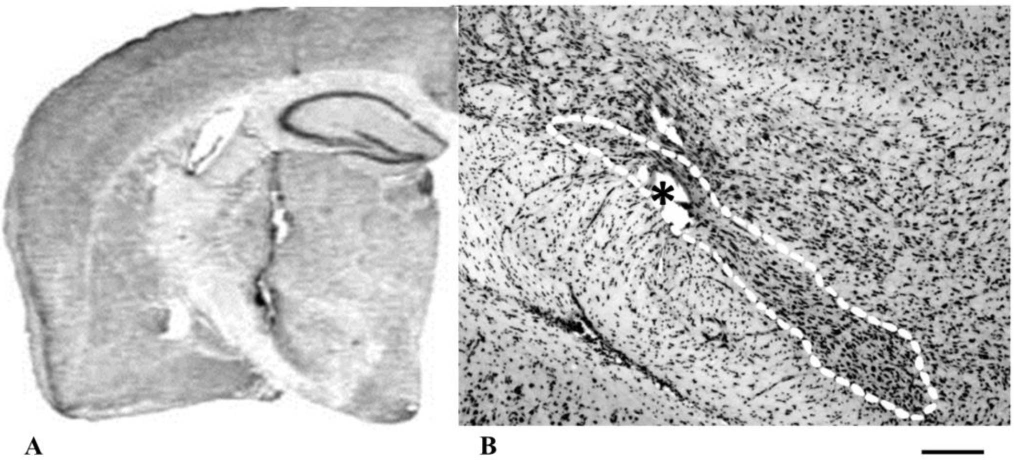

A B

Supplemental Figure 1: location of the DBS electrode tip within the STN. a) Low power photomicrograph illustrating the tract of the DBS electrode in the STN of a whole hemisphere of a mouse brain. b) High power photomicrograph illustrating the location of the DBS electrode tip (as indicated by a *) in the STN (as delineated by a dotted white line). Scale bar represents 0.2mm in b.
